# Highly Luminescent and Biocompatible P and N Co-Doped Passivated Carbon Nanodots for the Sensitive and Selective Determination of Rifampicin Using the Inner Filter Effect

**DOI:** 10.3390/ma13102275

**Published:** 2020-05-15

**Authors:** Baraa Al-Hashimi, Heshu Sulaiman Rahman, Khalid Mohammad Omer

**Affiliations:** 1 Department of Pharmacology, College of Medicine, University of Sulaimani, Sulaymaniyah 46002, Kurdistan, Iraq; baraa.abdulhameed@univsul.edu.iq; 2 Department of Physiology, College of Medicine, University of Sulaimani, Sulaymaniyah 46002, Kurdistan, Iraq; heshu.rhaman@univsul.edu.iq; 3 Department of Chemistry, College of Science, University of Sulaimani, Sulaymaniyah 46002, Kurdistan, Iraq

**Keywords:** rifampicin, carbon nanodots, inner filter effect, quenching, hydrothermal, cytotoxicity

## Abstract

The determination of rifampicin in pharmaceutical dosage forms using a rapid, sensitive, selective, biocompatible, and low-cost method is of vital importance in the pharmaceutical analysis field to ensure its concentration is within the effective range when administered. In this study, nitrogen-and-phosphorous-doped carbon nanodots (CNDs) were prepared using a single-step hydrothermal method with ciprofloxacin as the starting material. The CNDs showed a highly intense blue fluorescence emission centered at 450 nm, with a photoluminescence quantum yield of about 51%. Since the absorption of rifampicin was the same as the excitation spectrum of CNDs, inner filter effect (IFE) quenching occurred and it was used as a successful detection platform for the analysis of rifampicin in capsules. The detection platform showed a dynamic linear range from 1 to 100 μM (R^2^ = 0.9940) and the limit of detection was 0.06 μM (when S/N = 3). The average spike recovery percentage for rifampicin in the capsule samples was 100.53% (*n* = 5). Moreover, the sub-chronic cytotoxicity of CNDs was evaluated on healthy male mice (Balb/c) drenched with different amounts of CNDs (10 and 50 mg/kg). During this study period, no mortalities or toxicity signs were recorded in any of the experimental subjects. Based on the cytotoxicity experiment, the proposed nano-probe is considered safe and biocompatible.

## 1. Introduction

The inner filter effect (IFE) is a significant quenching process involving an energy conversion that is not associated with radiation; it has previously been regarded as an analytical error but has recently started to find its place in the analysis field [1]. It occurs due to the complementary overlap of the absorber UV absorption band with the fluorophore excitation and/or emission band, which results in the attenuation of fluorescence intensity [2]. Using nanosensors based on IFE does not require any kind of direct chemical interaction between the analyte and the sensor, which in turn offers advantages, such as simplicity, sensitivity, and low cost [3]. IFE has found success in the fields of pharmaceuticals, food, and environmental analysis for the sensing and determination of metal ions, drugs, and proteins [4,5,6].

Carbon nanodots (CNDs) are an advanced generation of fluorescent material that has drawn a lot of interest recently due to its desirable characteristics, such as water solubility, facile preparation, low synthesis cost, relatively high stability, and low toxicity [7]. Consequently, there has been an increasing interest in using carbon dots in many fields, such as bio-imaging [8], biomedicine [9], photocatalysis [10], drug delivery [11], and fluorescent probes [12]. CNDs are considered to be the most common form of carbon dots due to the simplicity of their synthesis and they can be further classified into carbon nanoparticles that do not possess a crystalline structure and carbon quantum dots that possess crystalline structure [13]. Moreover, the physicochemical characteristics of the synthesized CNDs can be further modified via surface modification and functionalization, which can be utilized to improve the selectivity and sensitivity for the desired analyte [14].

The selectivity of CNDs for a specific target is highly sought after for analytical applications, and it is affected by the chemical interaction between the analyte of interest and the chemical groups on the surface of the CNDs. The surface functional group and chemistry of the interaction can be tuned using different precursors, solvents, processing times, temperatures, and pHs [15]. Unfortunately, the theoretical modeling of the mechanism of the functionalization of CNDs with chemical groups is not well-understood yet; to obtain the optimum results, further experiments are required [16]. On the other hand, the enhancement of selectivity can be achieved via the utilization of CNDs with a high fluorescence quantum yield [17].

Rifampicin is an antibacterial drug that is used as a first-line treatment for tuberculosis, which is considered to be one of the top ten fatal diseases worldwide [18]. According to the World Health Organization (WHO), tuberculosis resulted in the deaths of 1.5 million people in the year 2018 alone, while 10 million individuals were infected by the pathogen [19]. The mechanism of action of rifampicin involves the inhibition of the bacterial-DNA-dependent RNA polymerase [20]. Toxicity from rifampicin can lead to serious adverse effects, such as hepatotoxicity, along with other less severe side effects, including arthralgia, fever, and allergic reactions [21]. As a result, it is vital to develop a simple, accurate, and fast method to detect and determine the level of rifampicin in samples to minimize the risk of toxicity. Currently, there are many methods for determining the amount of rifampicin, such as high-performance liquid chromatography (HPLC) [22], UV-vis spectroscopy [23], microbiological assays [24], and metal–organic frameworks [25]. These methods can provide high selectivity and good detection limits; however, they have the disadvantage of requiring high running costs, sample preparation, personnel skills, and tedious processing times. 

**Figure 1:**
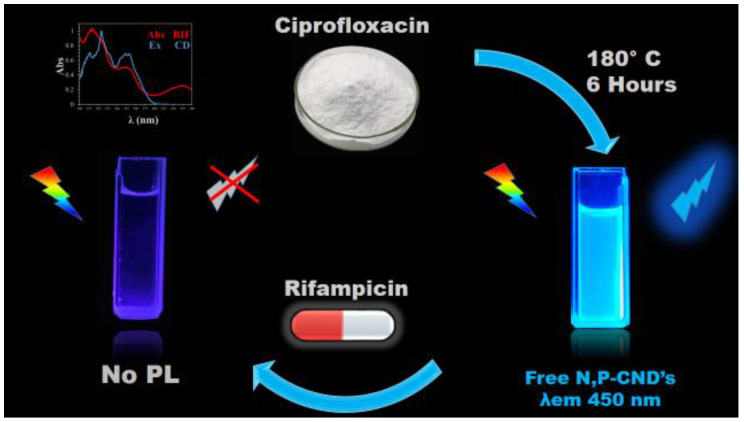
Schematic illustration of the hydrothermal preparation of the carbon nanodots (CNDs) and quenching using rifampicin due to the overlap of the rifampicin absorption spectra with the CNDs excitation spectra. PL: Photoluminescence.

Here, a particular nitro-fluoro drug, such as ciprofloxacin, was used as a carbon and nitrogen source to synthesize nitrogen-and-phosphorus-doped nanodots (CNDs), as shown in Figure 1. 

The hydrothermal approach was utilized, with phosphoric acid acting as the solvent and source of phosphorus, resulting in highly luminescent CNDs. The excitation spectra of the CNDs significantly overlapped with the absorption spectra of the drug rifampicin, which can be considered as a possible reason for the quenching. The preparations and running costs of this method were less than US$1 with a sufficient amount of CNDs to conduct thousands of tests as it has a high quantum yield and requires dilution of up to 150-fold, which is very convenient in comparison to standard protocols with HPLC that have running costs of up to US$400 per sample [26].

In summary, this study aimed to synthesize novel CNDs with a new source of carbon, characterize them by studying the morphological properties using functional groups analysis, apply the synthesized CNDs in the pharmaceutical analysis field for the determination of rifampicin in capsules, and finally study their biocompatibility and safety in vivo to set the stage for further applications, such as drug delivery and bio-imaging.

The CNDs were used as a sensitive tool for the detection of rifampicin, utilizing the IFE sensing platform. The proposed method was also used to assay rifampicin in real drug samples, which showed high selectivity, sensitivity, and biocompatibility.

## 2. Experimental Section

### 2.1. Chemicals

Standard rifampicin was acquired from Solarbio Life Sciences, Beijing, China. While the rifampicin capsules were obtained from a local drugstore. Pure ciprofloxacin (1-cyclopropyl-6-fluoro-1,4-dihydro-4-oxo-7-(1-piperazinyl)-3-quinolinecarboxylic acid) was imported from Abhilash Chemicals and Pharmaceuticals Private Limited, Madurai, India. Phosphoric acid was acquired from Sigma-Aldrich, St. Louis, MO, USA. All other chemicals and reagents used in this work were high-quality, analytical grade substances.

### 2.2. Synthesis of CNDs

The CNDs were prepared using a single-step solvothermal method. Briefly, 100 mg of ciprofloxacin was dissolved in 10 mL of concentered phosphoric acid, resulting in a clear solution that was then placed in a 25 mL Teflon-lined stainless-steel autoclave. The reactor was then heated at 180 °C for 6 h, which was then left to cool down at ambient temperature. Filtration was then applied to the latter solution using a 0.22 μm filter paper to eliminate any undissolved particles and impurities. For further purification, centrifugation at 12,000 rpm for 15 min was employed, followed by dialysis with a 1000 MWCO dialysis membrane for 24 h. The final solution containing the CNDs, which showed blue fluorescence when exposed to UV light, was stored at room temperature for further use. 

### 2.3. Instruments and Characterizations

The morphological properties of the CNDs were studied using transmission electron microscopy (TEM) on a TECNAI G2 F20 (Tecnai, AMES Laboratory, Ames, IA, USA) by casting one drop of the solution containing the CNDs on a copper mesh, then leaving it to dry. The X-ray diffraction (XRD) patterns were obtained using a PANalytical (PANalytical, Rigaku, Almelo, Netherlands). The Fourier-transform infrared (FTIR) spectra were recorded using a Thermo-Scientific Nicolet iS50 FTIR Spectrometer (Nicolet, Thermo-Scientific, Waltham, MA, USA) equipped with a diamond ATR unit. The analysis was achieved by fully drying the samples, then scanning over the range from 400 to 4000 cm^−1^ with a resolution of 8 cm^−1^. A Renishaw inVia Reflex (InVia, Renishaw, Gloucestershire, UK) equipped with a diode emitting at a wavelength of 785 nm and operating with a maximum energy output of 50 mW using Nd:YAG laser source was used to generate the Raman spectra. The spectral resolution was 2 cm^−1^ covering the range from 500 to 3000 cm^−1^. To further investigate the surface properties of the CNDs, X-ray photoelectron spectra (XPS) were measured using a Thermo-Scientific Escalab 250 XI (Escalab, Thermo-Scientific, Waltham, MA, USA) while utilizing CasaXPS^®^ software (version 2.3.22) to analyze the data. The UV-vis absorption spectra were measured using an Agilent Cary 60 Spectrophotometer (Cary 60, Agilent, Santa Clara, CA, USA) using quartz cuvettes with an optical distance of 1 cm. The fluorescence spectra were acquired on an Agilent Cary Eclipse Fluorescence Spectrophotometer (Cary Eclipse, Agilent, Santa Clara, CA, USA) equipped with an ultraviolet emitting xenon lamp. 

### 2.4. Preparation of the Rifampicin Samples

The content of 20 rifampicin capsules was collected and ground into a fine powder from which 98.90 mg was taken and dissolved in ethanol; then, filtration was applied using a 0.22 μm filter membrane to eliminate any impurities. This final solution was then further diluted with ethanol in a volumetric flask up to 100 mL and stored for further analytical procedures.

### 2.5. Analytical Assay

To conduct the rifampicin assay, 2.0 mL of the CND solution with a concentration of approximately 0.01 mg/mL was stirred with 2 mL of the diluted rifampicin solution at 25 °C and the pH was set at approximately 3 using an acetate buffer. The resulting solution was then homogenized using a vortex for 1 min. The fluorescence spectra were recorded afterward. 

### 2.6. Stability Experiments

Three main parameters of the stability of CNDs were investigated, including the thermo-stability, the effect of ionic strength, and the pH. The thermo-stability was evaluated by placing samples of CNDs in a water bath and heating over a range from 25 °C to 100 °C for 10 min. The ionic strength effect was investigated using a NaCl solution with different concentrations ranging from 0.1 M and up to 2.0 M. The pH of the CND aqueous solutions was modified over a wide range of 2 to 12 to study its effect.

### 2.7. Quantum Yield

Quinine sulfate in 0.1 M H_2_SO_4_ was used to calculate the relative phosphorescence quantum yield as the standard fluorescent material [27]. The following equation was used to determine the absolute values:

(1) $$\Phi_{X}=\Phi_{St}\left(\frac{M_{X}}{M_{St}}\right){\left(\frac{\gamma_{X}}{\gamma_{St}}\right)}^{2}$$

where *St* refers to the standard agent used and *X* refers to the sample. Φ is the quantum yield, *γ* stands for the refractive index assay, and *M* is the gradient of the spectrum plot. To maintain linearity and minimize deviations, both the sample and standard were diluted to obtain the absorbance spectra below 0.1 arbitrary unit.

### 2.8. Animals

Adult male mice weighing 20–22 g and aged 8–10 weeks, donated by the College of Educational Science, Department of Biology, University of Sulaimani, were used in this study. Before the commencement of the experiment, the mice’s adaptation to laboratory conditions was achieved by placing them in straw wood litter polypropylene cages for 7 days with tap water and standard mice pellets; the room had an ambient temperature of about 25 ± 2.0 °C with a regular light/dark cycle. The College of Medicine, University of Sulaimani, issued the ethical consent for carrying out this study.

### 2.9. Sub-Chronic Toxicity Assessment

Three groups, each consisting of five mice, were allocated for the assessment of sub-chronic toxicity. Tap water only was administered to the first group, which represented the control negative, whereas the second and third groups were given a low dose (LD: 10 mg/kg CNDs) and a high dose (HD: 50 mg/kg CNDs), respectively. Constant dosing in the early morning was conducted using a forceful stainless steel needle for 4 consecutive weeks, which was proceeded by a 12 h starvation period each time [28]. 

### 2.10. Clinical Behaviour and Body Mass Analysis

All test subjects were evaluated in terms of abnormal behavior, diet consumption, and signs of toxicity two times a day during the experimental period. Measurements of the test subject’s body masses were conducted on days 0, 14, and 28 using a Sartorius digital weighing scale. 

### 2.11. Hematological and Blood Biochemistry Assay

At the end of the study and before sacrificing of the animals, approximately 1–1.5 mL of blood was drawn via cardiac piercing while anesthetizing the test subjects; half of the drawn blood was kept in tubes with an anticoagulant and used for hemograms including complete blood count (CBC) and packed cell volume (PCV) using a colter counter, while the other half was used for serum collection via centrifugation and for conducting blood biochemistry, including liver and kidney function tests using optimized diagnostic kits (Roche Diagnostic GmbH, Indianapolis, IN, USA) and analyzed using a 902 Hitachi automatic analyzer (Hitachi LTD, Chiyoda, Japan).

### 2.12. Histopathology

Directly after sacrificing each animal under general anesthesia using a mixture of xylazine and ketamine, samples from the liver and kidney were extracted, cleaned using a phosphate buffer solution (PBS), reduced in size, and placed in a fresh formalin solution (10%) for at least 48 h. Later on, sections of tissue were placed in test cassettes to undergo gradual dehydration via an automated tissue processor. After that, dehydrated samples were placed in paraffin wax to obtain blocks. Subsequently, a semi-automated microtome was used for tissue sectioning, and sections were fixed on glass slides using a hot plate. Different concentrations of ethanol were used to rehydrate the slides, which were then washed with tap water. The slides were stained with Harris’s hematoxylin and eosin (H&E) to allow for examination under a normal light microscope (Olympus, Japan).

## 3. Results and Discussion

### 3.1. Morphology and Surface Composition

**Figure 2:**
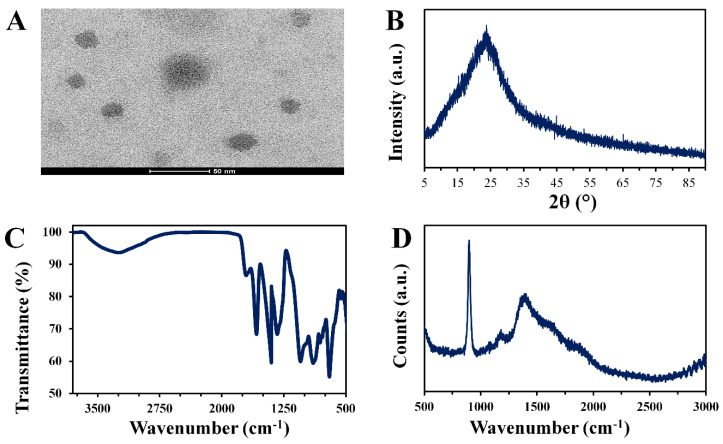
(**A**) TEM image, (**B**) XRD pattern, (**C**) FTIR spectra, and **(D**) Raman spectra of the CNDs.

The size and shape of the CNDs were studied using TEM (Figure 2A).

The images of the TEM demonstrated that the CNDs had a spherical shape with an average size of 10 nm. The size of the particles is of extreme importance as a high quantum yield is achieved with a size of approximately 10 nm, broadening the applications of the CNDs [29]. According to the literature, carbon nanodots with a size of less than 17 nm are generally circular, while particles with a size of more than 17 nm tend to become polygonal [30]. Furthermore, the particle size can be reduced with increasing reaction time, increasing reaction temperature, and adjusting the precursor-to-solvent ratio [31]. The XRD spectra (Figure 2B) showed the characteristic broad peak at 2θ = 23.8 Å, which corresponded to the graphite structure of the CNDs [32]. 

FTIR revealed more information about the functional groups of the CNDs (Figure 2C). It was noticed that a very broad band was observed at 3300 cm^−1^, which originated from the hydroxyl group of the carboxyl present on the surfaces of the CNDs [33]. A band typical for C=O stretching was noticed at 1700 cm^−1^, while C=C stretching in the shape of a medium peak was observed at 1610 cm^−1^ [34]. Furthermore, a peak typical for C–O stretching was noticed at 1050 cm^−1^ and a band related to C–H bending was observed at 900 cm^−1^ [35]. More detailed information about the functional groups of the CNDs was obtained from the Raman spectral analysis (Figure 2D). A strong band was centered at 900 cm^−1^, which was attributed to C–C stretching [36]. Deformation vibrations of a hexagonal ring or amorphous carbon (D band) were represented by a peak at 1360 cm^−1^, while a shoulder was observed at 1590 cm^−1^, which was related to the sp^2^ graphitic domain (G band) [37]. 

**Figure 3:**
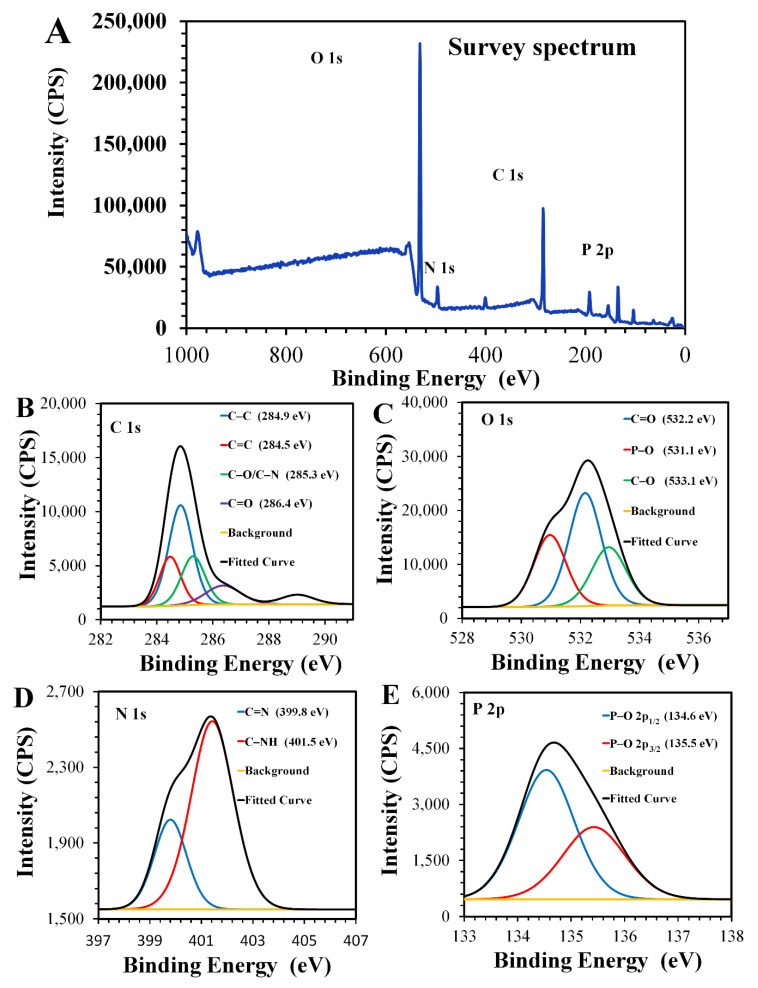
High-resolution XPS spectra of the CNDs: (**A**) survey spectra of CNDs and (**B**–**E**) deconvoluted XPS spectra for all elements in the CNDs.

X-ray photoelectron spectroscopy (XPS) was used to gain more information about the composition of the CNDs. Four peaks were observed on the survey XPS spectra: C 1s (285 eV), O 1s (532 eV), N 1s (400 eV), and P 2p (134 eV) (Figure 3A). 

The deconvoluted C 1s demonstrated four peaks at 284.5 eV, 284.9 eV, 285.3 eV, and 286.4 eV, which corresponded to C–C, C=C, C–O/–N, and C=O, respectively (Figure 3B) [38]. The high resolution O 1s spectra (Figure 3C) showed three peaks: 531.1 eV (P–O), 532.2 eV (C=O), and 533.1 eV (C–O) [39]. Figure 3D shows the deconvoluted N 1s spectra in which two peaks were observed, the first was at 399.8 eV, which indicated C=N, and the second was at 401.5 eV, which indicated C–NH [40]. The deconvoluted P 2p spectra showed two peaks at 134.6 eV and 135.5 eV, which indicated P–O 2p_1/2_ and P–O 2p_3/2_, respectively (Figure 3E) [41]. The presence of nitrogen and phosphorus peaks indicates that the CNDs were doped with N and P.

### 3.2. Optical Properties

**Figure 4:**
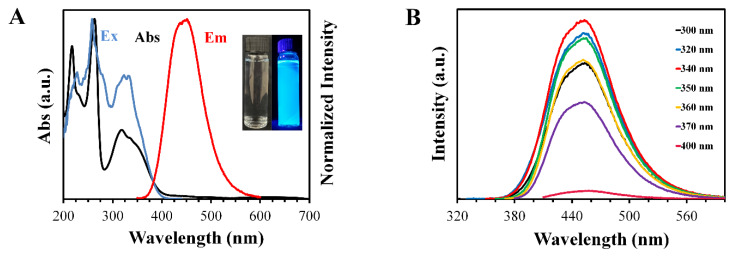
(**A**) Excitation (blue), absorption (black), and emission (red) spectra of the CNDs (inset: digital picture of the CND solution under visible light (left) and ultraviolet light (right)). (**B**) PL emission of CNDs with various excitation wavelengths.

The optical properties of the CND were studied using UV-vis absorption spectra and fluorescence emission/excitation spectra. The absorption spectra shown in Figure 4A displayed a sharp peak at 220 nm, which could have been the result of the π to π* transition of the C=C bonds [42]. 

A broad peak was observed at 320 nm, which may have originated from the n to π* transition [43].

Exciting the CNDs at 340 nm resulted in the highest intensity of emission in the fluorescence spectra, which was observed at around 450 nm. The CNDs showed wavelength-independent fluorescence emissions, where changing the excitation wavelength did not result in any significant change in the position of the emission peak (Figure 4B), which was attributed to the full passivation of the surface of the CNDs with the functional group [44], or it may have been some molecular fluorophores that formed and had attached to the surface of the CNDs [44,45]. Either way, this phenomenon is unclear and is debated in the literature. 

### 3.3. Stability of the CNDs

**Figure 5:**
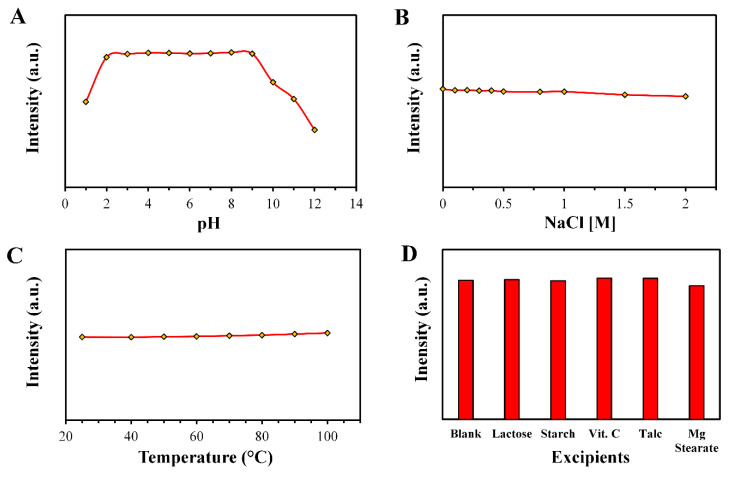
The effect of pH (**A**), ionic strength (**B**), temperature (**C**), and excipients (**D**) on the fluorescence intensity of the CNDs.

Variations in the fluorescence intensity were measured with changing pH, ionic strength, and temperature of the aqueous solutions of CNDs to study their stability profile. Although placing the CNDs in highly acidic (less than 2) or basic pH (higher than 9) resulted in decreasing the fluorescence intensity, the samples showed high stability over a broad pH range of 2 to 9, which could be attributed to the chemical changes in the surface of the CNDs due to protonation and deprotonation [46,47], as shown in Figure 5A. 

High ionic strength stability was observed after adding NaCl to the CND solution at various concentrations of up to 2 M (Figure 5B). Regarding the thermostability, no alteration in the photoluminescence intensity was noted after heating the samples of CNDs for at least 10 min up to 100 °C (Figure 5C). This could be attributed to the surface composition, which was rich with carboxyl and hydroxyl groups, as well as the presence of an amine group that could improve the stability of the CNDs by stabilizing the particle aggregation and diffusing in the fluorescence centers, protecting them from degradation while being in a high-temperature environment [48]. Consequently, the CNDs showed high stability under various conditions, which assured the versatility of applications.

### 3.4. Selectivity of the CNDs

High concentrations of inactive pharmaceutical ingredients (the excipients) were mixed with the aqueous solution of CNDs to investigate the interference effect on the fluorescence intensity (Figure 5D). The common excipients with rifampicin were lactose, starch, vitamin C, magnesium tartarate, and talc. Negligible changes were observed in the intensity of the fluorescence with the presence of the additives, which could be attributed to random error. High selectivity boosted the value of the CNDs in the assay of the active ingredient of rifampicin in the rifampicin capsules. 

### 3.5. Mechanism of Quenching

**Figure 6:**
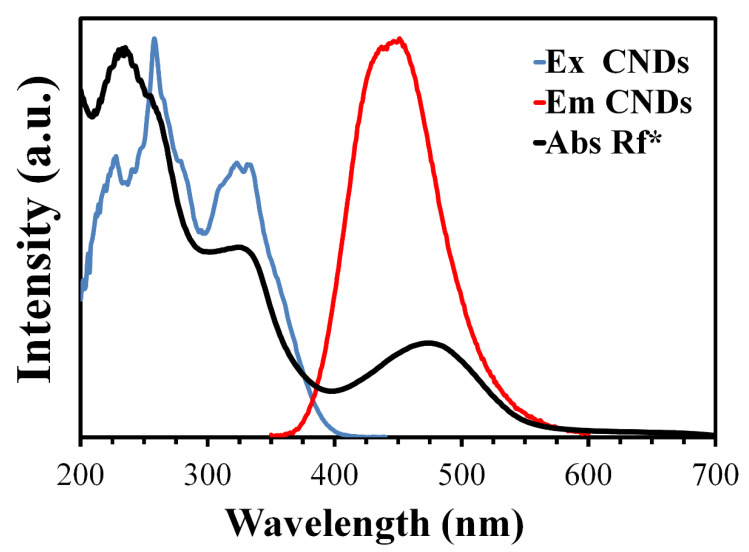
Absorption spectra of rifampicin, and the excitation and emission spectra of the CNDs. Rf* stands for rifampicin.

To provide a possible theory for the quenching mechanism, the overlapping between the rifampicin absorption spectrum and the CNDs excitation/emission spectra was studied. As shown in Figure 6, the peaks of both the excitation and emission spectra of the CNDs overlapped with the absorption spectra of rifampicin, which resulted in a decreased intensity of the fluorescence of the CNDs due to the absorption of radiation by rifampicin [49]. 

Since both the excitation and emission spectra of the CNDs were affected by the absorption spectra of rifampicin, this means the type of inner filter effect involved both the primary and secondary IFE [50].

### 3.6. Assay of Rifampicin Capsules

**Figure 7:**
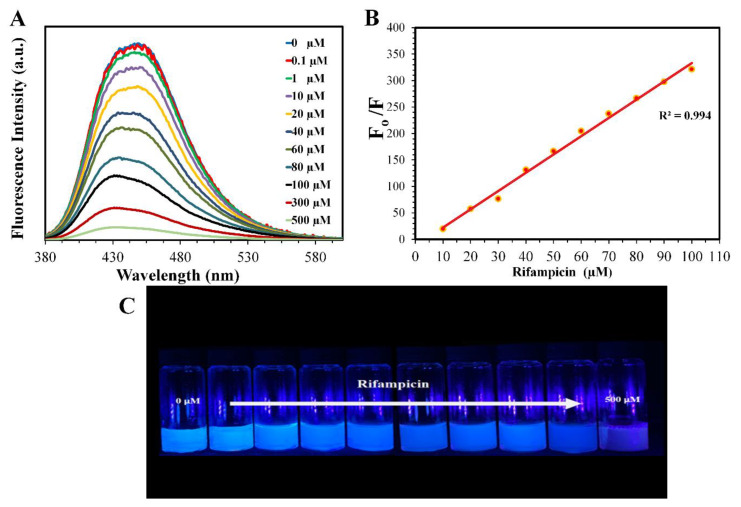
(**A**) Fluorescence spectra of the CNDs with the addition of different concentrations of rifampicin. (**B**) Graph of F_0_/F versus the concentration of rifampicin, where F_0_ is the fluorescence intensity when there was no rifampicin and F is the fluorescence intensity when rifampicin was added. (**C)** The digital micrograph showing the addition of various concentrations of rifampicin to the CND solutions.

The quenching effect resulting from the addition of rifampicin to the aqueous solution of the CNDs was exploited to determine the capsules’ active pharmaceutical ingredient content. A calibration graph was established using a series of standard solutions prepared from certified reference material (Figure 7); this was achieved by plotting the obtained fluorescence against the concentration (R^2^ = 0.9940), where the linear range for the response was from 1 µM to 100 µM. 

The detection limit was 0.06 µM (signal-to-noise ratio = 3), while the quantification limit was 0.207 µM (signal to noise ratio = 10). Standard addition and external standard methods were used to quantify the content of the capsules and determine the recovery percentage (Table 1). 

The average content of rifampicin in the capsules was 301.6 mg, which represented 100.53% of the indicated amount; this result falls within the acceptable limits of not less than 92.5% and not more than 107.5% of the indicated amount, as stated by the British Pharmacopeia (BP) [55]. Based on the results, the proposed method can be used as a probe for the detection and assay of rifampicin in pharmaceutical products with high selectivity and sensitivity. In comparison to other methods from the literature, the current method provided a detection limit that was superior to most of them (Table 2).

### 3.7. Cytotoxicity

None of the animals in either treatment group showed any signs of toxicity, such as separation from other animals in the same cage, stunting, and shedding hair, or erratic behaviors, such as loss of appetite, fierceness, and cannibalism. Simultaneously, a continuous increase in body weight was observed in test subjects of all groups throughout the experiment, which indicated the lack of any negative effect of the CNDs on the growing capability of the mice (Table 3). 

The variation between the testing dates was analyzed using a one-way ANOVA post hoc comparison test. The results showed a significant increase in the body mass throughout the study duration (*p* < 0.05), with the control group value being comparable to both treated groups.

Furthermore, the hepatic and renal function tests for both treated and untreated groups did not show any significant differences (*p* > 0.05) (Table 4). 

The date have been analysed with post hoc comparison test one way ANOVA. The data did not show any significant differences (*p* > 0.05) in hepatic and renal function results after 4 weeks of administration of CNDs amongst all studied groups. ALP: alkaline phosphatase, ALT: alanine aminotransferase, AST: aspartate aminotransferase.

Furthermore, the results showed normal values for the total red blood cells RBC, total white blood cells WBC, haemoglobin Hb, packed cell volume PCV, and platelets PLT in the tested blood (Table 5). 

The hemogram results were analyzed using a one way ANOVA post hoc comparison test. The data did not show any significant differences (*p* > 0.05) in the hemogram results after 4 weeks of administration of CNDs amongst all studied groups.

Thus, polycythaemia or anaemia, fluctuations in platelets and WBC counts, or pancytopenia were not anticipated in the tested subjects, which excluded the possibility of damage to bone marrow.

**Figure 8:**
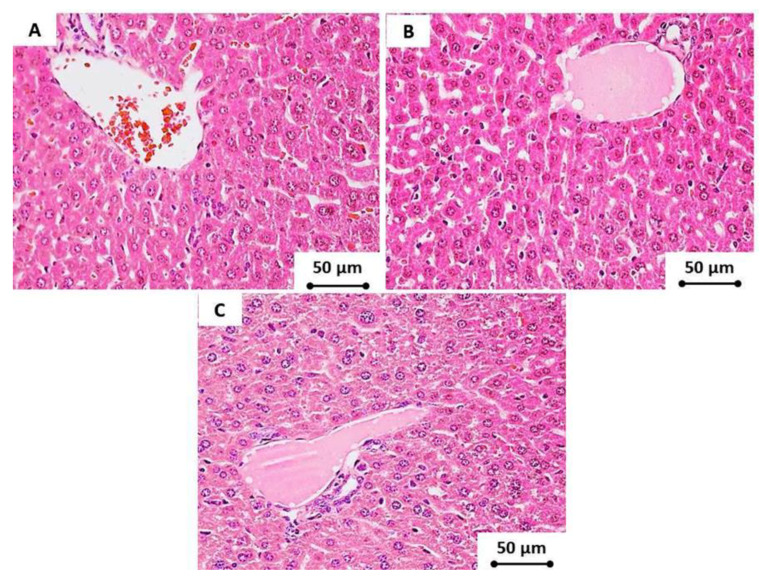
The hepatic tissue (at 400× magnification) of the tested subjects that were administered (**A**) tap water, (**B**) low doses of CNDs (10 mg/kg), and (**C**) high doses of CNDs (50 mg/kg) after 4 weeks. There were no pathological changes in any of the tested groups.

Moreover, microscopic examinations of the hepatic tissue samples of the animals administered with CNDs showed a normal, undilated central vein; the surrounding hepatocytes exhibited no congestion in the portal veins and hepatic sinusoids; no infiltration of inflammatory cells was observed; and there was no collagen proliferation in between the newly formed bile ducts at the portal area. Furthermore, there were no degenerative changes or apoptosis of the hepatocytes (Figure 8). 

**Figure 9:**
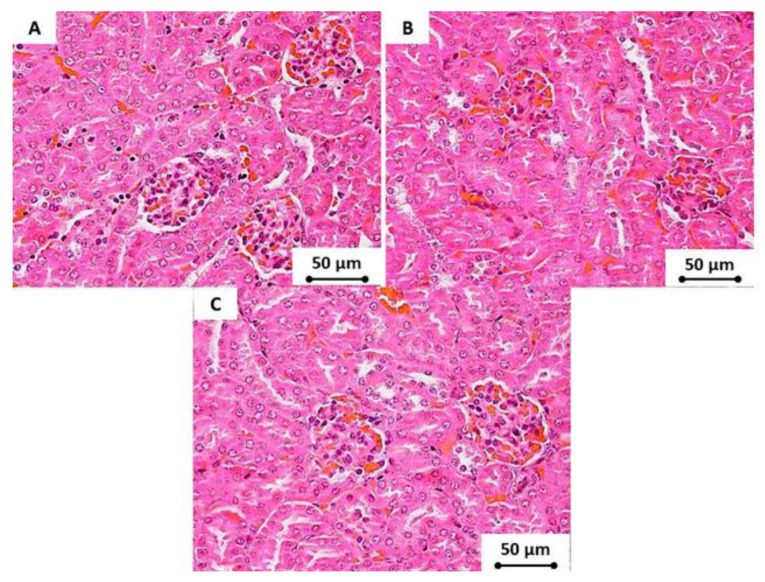
The renal tissue (at 400× magnification) of the tested subjects that were administered (**A**) tap water, (**B**) low doses of CNDs (10 mg/kg), and (**C**) high doses of CNDs (50 mg/kg) after 4 weeks. There were no pathological changes in any of the tested groups.

Additionally, there was no histological alteration in the renal tissues of tested subjects for all groups, and the glomerular tuft had a natural appearance. The cross-sections of the glomeruli exhibited a normal structure in all treated mice without any congestion, dilatation, swelling, or vacuolization in the lining endothelium that is usually associated with inflammatory cell infiltration. Furthermore, a normal appearance was displayed by the nephrons without degeneration or dilatation. Additionally, no renal cast formation was exhibited in the tubular lumen and cystic tubular dilatation (Figure 9). 

As a result, the lethal dose (LD) of CNDs must be a concentration higher than 50 mg/kg.

## 4. Conclusions

In summary, an IFE sensing probe was successfully applied for the identification of rifampicin in capsules using new highly photoluminescent nitrogen-and-phosphorus-doped CNDs. The proposed method offers advantages in terms of its simplicity and low-cost production of the CNDs. Common excipients of the rifampicin capsule did not interfere with the nano-probe. Moreover, the CNDs showed a low LOD 0.06 µM, which was below the level permitted level of rifampicin in capsules. Consequently, the prepared CNDs show great potential for use as an alternative for rifampicin analysis in the pharmaceutical field. We concluded that CNDs displayed no cytotoxic effects on internal organs, renal and hepatic function tests, and blood parameters in experimental animals when given orally for four consecutive weeks.

## Figures and Tables

**Table 1:** Review of various approaches used for rifampicin assays.

Material	Method	LOD (μM)	Linearity Range (μM)	Reference
Nickel nanoparticles	Voltammetric	2.6	5–500	[51]
C18 monolithic column	HPLC	0.2	5–200	[52]
Peroxomonosulfate-Co (II)	Chemiluminescence	0.008	0.06–1.21	[53]
Carbon nanodots	Fluorometry	0.15	0.52–59.5	[54]
Carbon nanodots	Fluorometry	0.06	1–100	This work

**Table 2:** Rifampicin assays and the determination of recovery percentage using CNDs (n = 5).

Sample No.	Actual Amount (mg)	Measured Amount (mg)	Recovery (%)	RSD (%)
**1**	298.39	302.4	100.80	1.03
**2**	297.98	302.2	100.73	0.98
**3**	297.88	298.8	99.60	0.18
**4**	298.26	304.4	101.47	1.50
**5**	298.38	300.2	100.07	0.51

**Table 3:** Body mass of tested subjects throughout study duration (mean ± SD, n = 5).

Animal Group	Day 0	Day 14	Day 28
Control	20.15 ± 1.3	25.0 ± 0.8	29.8 ± 1.0
Low dose	20.05 ± 1.2	24.15 ± 0.45	27.9 ± 1.5
High dose	20.3 ± 1.5	25.45 ± 0.75	28.66 ± 0.8

**Table 4:** Hepatic and renal function parameters of the test subjects (mean ± SD, n = 5).

Animal Group	ALP (U/L)	ALT (U/L)	AST (U/L)	Urea (mmol/L)	Creatinine (µmol/L)
Control	110.5 ± 0.3	60.5 ± 1.0	145.5 ± 1.3	7.9.0 ± 0.5	30.5 ± 0.05
Low dose	111.75 ± 0.5	61.63 ± 1.5	144.2 ± 0.75	8.2 ± 1.2	30.1 ± 1.7
High dose	112.5 ± 0.65	61.07 ± 1.2	145.8 ± 0.7	8.0 ± 1.6	30.5 ± 1.9

**Table 5:** Results of hemogram for test subjects (mean ± SD, n = 5).

Animal Group	Total RBC (× 10^12^/L)	Hb (g/L)	PCV (L/L)	PLT (× 10^5^/L)	Total WBC (× 10^3^/µL)
Control	8.3 ± 0.2	139 ± 1.3	0.39 ± 0.25	6.6 ± 0.85	2.6 ± 0.1
Low dose	8.4 ± 1.5	141 ± 1.7	0.41 ± 0.45	6.4 ± 0.33	2.55 ± 0.55
High dose	8.2 ± 0.75	142 ± 1.45	0.40 ± 0.3	6.3 ± 0.11	2.45 ± 0.65
